# Trial for Malpractice. Tims vs. White. Case of Fractured Femur

**Published:** 1848-08

**Authors:** 


*Trial for Malpractice. Tims* vs. *White. Case of Fractured Femur.*

This suit was instituted nearly five years ago, the plaintiff charging the defendant with inattention and unskillfulness in the management of the case, in consequence of which the femur was deformed, and the usefulness of the limb impaired. It was tried three times, the jury on the two first trials not being able to agree upon a verdict. On the last trial, the jury returned instantly a verdict for the defendant.

A pretty full report of the trial has been furnished us, and although it occupies considerable space, we think it deserves to be published, and perused, for several reasons. This is, in the first place, due to the parties concerned. The fact alone of being sued for malpractice, is apt to convey an imputation upon the professional character of the defendant, which is only to be removed by giving publicity to all the facts which the trial discloses. By reference to these facts also, the reader is able to form an opinion as to the motives which have actuated the plaintiff, in endeavoring to recover, at law, damages for supposed grievances.

The trial is interesting in a scientific point of view. The question at issue, as to the cause of the shortening and deformity in the case, involves practical considerations of importance to the surgeon. It is interesting also from the fact that it embraces the testimony of several eminent practitioners, not only as respects the facts of this particular case, but concerning the treatment, etc., of fractures generally. The reader will observe that among the medical witnesses examined at the three trials, were four well-known teachers of Surgery.

And as a contribution to the annals of trials for mal-practice, it possesses value. It is certainly a significant commentary on suits of this character, that this suit, which had dragged its slow length along for the space of five years, was at length decided by the jury on the first balloting. The commentary is rendered still more significant by the following particulars, which it may not be improper to mention, in this connection:—At the first trial, it was generally understood, a majority of the jury were in favor of a verdict for the plaintiff. At the second trial, a *struck jury* was obtained; i. e. forty-eight intelligent persons were selected by the County clerk; of these, each party had the privilege of striking off the names of twelve, and of the remaining twenty-four, the names of twelve were drawn by ballot. A jury obtained in this way, is presumed to embrace more intelligence than juries procured in the ordinary mode. Nevertheless, at the second trial, the struck jury failed to agree. At the third trial, as already remarked, a verdict was returned for the defendant without any consultation, the jury being out only long enough to express their unanimity by balloting. The facts disclosed by each of the trials, were, in all material respects, the same, and by perusing the report, the medical reader will be able to form an opinion as to their character. Whatever this opinion may be, the history of the suit illustrates the glorious uncertainty of the law in cases of this description. Should it appear, from an examination of the testimony, that the plaintiff had no just ground for complaint, the reader cannot fail to arrive at the conclusion that it is not always an easy matter for the defendant, under such circumstances, to achieve justice; and that suits for mal-practice cannot be considered matters of trifling moment, as regards loss of time and money, and mental inquietude, notwithstanding the defendant comes off in the end triumphant.

