# Erratum

**Published:** 1848-08

**Authors:** 


*Erratum,*—On the 193d page, 5th line, for "*clearly* written book" read *cleverly* written book.

